# Phenotypic and genotypic analysis of *Helicobacter pylori* resistance patterns, molecular characteristics, and CYP2C19 polymorphisms in Nanjing: a retrospective cross-sectional study

**DOI:** 10.3389/fcimb.2026.1852534

**Published:** 2026-06-23

**Authors:** Haocheng Wang, Chao Li, Ruolin Peng, Xiujuan Wang, Jiahuan Gao, Zhenyu Zhang

**Affiliations:** Department of Gastroenterology, Nanjing First Hospital, Nanjing Medical University, Nanjing, China

**Keywords:** antibiotic resistance, CYP2C19 polymorphisms, genotypic testing, *Helicobacter pylori*, Nanjing

## Abstract

**Background:**

The continued rise in antibiotic resistance of *Helicobacter pylori* (*H. pylori*) poses a major challenge to clinical eradication therapy. The resistance patterns and phenotype-genotype concordance of *H. pylori* in the Nanjing region have not been systematically reported in recent years.

**Methods:**

Data from 7227 enrolled patients at Nanjing First Hospital (2018–2024) were retrospectively analyzed. Among these, 5729 patients were culture-positive for *H. pylori* and underwent phenotypic antimicrobial susceptibility testing for six antibiotics. Genotypic testing for resistance-associated mutations was performed on a subset of these patients. The remaining 1498 patients, although *H. pylori*-positive by other diagnostic methods, were culture-negative and were excluded from resistance analyses.

**Results:**

Metronidazole showed the highest resistance rate at 84.45% (4838/5729, 95% CI: 83.50–85.37), followed by clarithromycin at 50.01% (2865/5729, 95% CI: 48.71–51.31) and levofloxacin at 38.59% (2211/5729, 95% CI: 37.33–39.87). Resistance rates to amoxicillin at 0.51% (29/5729, 95% CI: 0.34–0.73), furazolidone at 0.19% (11/5729, 95% CI: 0.10–0.34), and tetracycline at 0.07% (4/5729, 95% CI: 0.02–0.18) were very low. Annual trend analysis showed that the metronidazole resistance rate remained above 92% since 2020, reaching 98.68% in 2024. Clarithromycin resistance increased from 35.80% to 60.14% and then declined slightly, while levofloxacin resistance rose from 20.99% to 44.59% before reaching a plateau. Genotypic testing for clarithromycin and levofloxacin showed excellent concordance with phenotypic susceptibility (Kappa values: 0.9046 and 0.9187, respectively; both P<0.001). Resistance mutations were highly concentrated in the *23S rRNA* gene at the A2143G locus (98.93%) for clarithromycin and in the *gyrA* gene at the N87K locus (52.54%) for levofloxacin. Kappa values for amoxicillin, tetracycline, furazolidone, and metronidazole were all below 0.2 and not statistically significant. CYP2C19 genotyping revealed that 43.89% of patients were extensive metabolizers, 44.26% intermediate metabolizers, 11.67% poor metabolizers, and 0.18% ultrametabolizers.

**Conclusion:**

Metronidazole, clarithromycin, and levofloxacin are no longer suitable as empirical first-line agents in Nanjing. Amoxicillin, furazolidone, and tetracycline remain reliable options. Genotypic testing for clarithromycin and levofloxacin resistance is highly concordant with phenotypic results and suitable for guiding individualized therapy. CYP2C19 genotyping supports personalized acid suppression strategies. Regimens containing amoxicillin, furazolidone, or tetracycline should be prioritized, with resistance gene testing recommended before using clarithromycin or levofloxacin.

## 1 Introduction

*Helicobacter pylori* (*H. pylori*) is a Gram-negative spiral-shaped bacterium that colonizes the human gastric mucosa. Numerous studies have confirmed that *H. pylori* infection is a causative factor for several diseases, including peptic ulcer disease, chronic gastritis, gastric cancer, and gastric mucosa-associated lymphoid tissue lymphoma (Malfertheiner et al., 2022; Zhou et al., 2022). The World Health Organization classified *H. pylori* as a Group I carcinogen for gastric cancer in 1994 (Møller et al., 1995). The most recent Chinese guideline identifies *H. pylori* infection as the most important modifiable risk factor for gastric cancer in China (Zhou et al., 2022). Two large cohort studies (Li et al., 2019; Chiang et al., 2021) have shown that eradication of *H. pylori* infection reduces the risk of gastric cancer by approximately 50%. Nevertheless, the global prevalence of *H. pylori* infection remains as high as 50% (Li et al., 2023), indicating a serious challenge for prevention and control. Therefore, timely detection and eradication of *H. pylori* infection are of great significance for reducing the burden of gastric cancer.

Bismuth-containing quadruple therapy is currently recommended as a first-line eradication regimen for *H. pylori* by clinical guidelines in many countries and is widely used in clinical practice (Zhou et al., 2022; Malfertheiner et al., 2022; Chey et al., 2024). However, the success rate of *H. pylori* eradication has been declining due to the increasingly serious problem of antimicrobial resistance, posing a significant challenge to clinical treatment. Data show that from 2018 to 2020, the primary resistance rates of *H. pylori* to clarithromycin, metronidazole, and levofloxacin in China reached 37.00%, 87.87%, and 34.21%, respectively, while the secondary resistance rates were as high as 76.93%, 93.48%, and 61.58%, all indicating high levels of resistance (Zhong et al., 2021). Therefore, selecting susceptible antibiotics is crucial for successful *H. pylori* eradication.

Antimicrobial resistance in *H. pylori* is currently widespread and shows considerable geographical variation (Xie and Lu, 2015). Therefore, further screening of the local antimicrobial resistance profile of *H. pylori* is essential for developing region-specific prevention and treatment strategies and for reducing infection rates. As the capital of Jiangsu Province, Nanjing has a large and diverse population. However, most existing epidemiological data on *H. pylori* infection and resistance in this region are outdated and lack up-to-date information reflecting recent trends, which limits the development of precise prevention and treatment strategies. To obtain reliable and representative up-to-date epidemiological data, it is first necessary to identify a simple and accurate method for detecting *H. pylori* resistance.

Phenotypic resistance testing following *H. pylori* culture is currently the gold standard for non-invasive resistance detection. This method allows comprehensive evaluation of susceptibility to six antibiotics (clarithromycin, levofloxacin, metronidazole, amoxicillin, tetracycline, and furazolidone), offering reliable results and broad coverage. Although it has limitations such as a long culture period and high requirements for equipment and environmental conditions, these do not diminish its value in terms of accuracy and guidance for clinical medication (Atkinson and Braden, 2016). With the advancement of molecular biology techniques, research into the molecular mechanisms of *H. pylori* resistance has deepened. Genotypic resistance testing evaluates *H. pylori* susceptibility by detecting mutations in resistance-related genes: *23S rRNA* for clarithromycin, *gyrA* for quinolones, *PBPs* for amoxicillin, 1*6S rRNA* for tetracycline, *porD* for furazolidone, and *rdxA*, *frxA*, and *frxB* for metronidazole. Among these molecular methods, polymerase chain reaction (PCR) using gastric mucosal specimens is rapid, timely, and highly accurate, and has been recommended by relevant guidelines for *H. pylori* genotypic resistance testing (Malfertheiner et al., 2022). Furthermore, Chen et al. compared susceptibility-guided therapy with molecular-guided therapy in 880 *H. pylori*-infected patients and found that molecular-guided therapy was non-inferior to susceptibility-guided therapy (Chen et al., 2023). Therefore, PCR-based genotypic resistance testing using gastric mucosal specimens is now more widely and efficiently applicable in clinical practice. This method partially overcomes the long turnaround time and high technical demands of traditional culture-based testing, improving the convenience and timeliness of clinical resistance screening while maintaining accuracy.

In summary, this study provides an updated analysis of *H. pylori* resistance patterns in Nanjing, China. It systematically characterizes the current status and temporal trends of phenotypic and genotypic resistance to six commonly used antibiotics (clarithromycin, metronidazole, levofloxacin, amoxicillin, tetracycline, and furazolidone), and evaluates the concordance between phenotypic and genotypic testing results. Additionally, the study analyzes the distribution and dynamic evolution of CYP2C19 polymorphisms in this region. These findings offer a scientific basis and practical reference for rapid diagnosis, resistance surveillance, and individualized treatment of *H. pylori* infection in Nanjing.

## 2 Materials and methods

### 2.1 Study overview

This study is a retrospective, single-center, cross-sectional epidemiological investigation. We retrospectively reviewed the medical records of outpatients who underwent *H. pylori* culture and genetic testing at the Gastroenterology Department of Nanjing First Hospital between 2018 and 2024. The aim was to characterize the resistance patterns of *H. pylori* in the Nanjing region, with a focus on the phenotypic and genotypic resistance to amoxicillin, clarithromycin, metronidazole, levofloxacin, tetracycline, and furazolidone, as well as the concordance between the two testing methods. Notably, not all enrolled patients had both phenotypic and genotypic testing results available. The specific sample sizes for each analysis are detailed in the Results section. The study was approved by the Medical Ethics Committee of Nanjing First Hospital Affiliated to Nanjing Medical University (Approval No. KY20260108-KS-10), which granted a waiver of informed consent for participants. For patients under 18 years of age, written informed consent was obtained from both the minor patients and their parents or legal guardians prior to these clinical procedures, in accordance with standard clinical practice and hospital regulations. The study is also registered with ClinicalTrials.gov (Registration No. NCT07455448; https://register.clinicaltrials.gov/).

### 2.2 Study participants

A total of 7,910 initial medical records were retrieved from the electronic medical record system and laboratory information system of Nanjing First Hospital. These records were from outpatients who underwent *H. pylori* culture-based phenotypic resistance testing and genetic testing at the Gastroenterology Department between January 1, 2018, and December 31, 2024. After screening according to pre-defined inclusion and exclusion criteria, 683 records that did not meet the criteria were excluded, and a total of 7,227 participants were ultimately included in the analysis. Of these, 5729 (79.27%) were culture-positive for *H. pylori* and underwent phenotypic antimicrobial susceptibility testing. The remaining 1498 patients who were culture-negative were excluded from the resistance analysis. Furthermore, genotypic testing was performed on a subset of the culture-positive patients, and the numbers of paired phenotype-genotype samples differed for each antibiotic.

#### 2.2.1 The inclusion criteria included:

1. Patients who visited the Gastroenterology Department of Nanjing First Hospital and underwent gastroscopy as well as *H. pylori* culture-based phenotypic resistance testing and genetic testing between 2018 and 2024, including minor patients.
2. Positive for *H. pylori* by at least one diagnostic method (such as ^13^C-urea breath test, rapid urease test, or histopathological examination).
3. Patients who provided general written informed consent for gastroscopy.

#### 2.2.2 The exclusion criteria were:

1. Use of antibiotics, proton pump inhibitors, H2-receptor antagonists, or bismuth agents within four weeks prior to gastroscopy.
2. History of gastric surgery.
3. Other factors deemed by the investigator to render the participant unsuitable for inclusion.

Enrollment into this study required participants to meet the inclusion criteria, including a positive result for *H. pylori* by at least one diagnostic method (¹³C-urea breath test, rapid urease test, or histopathological examination). However, phenotypic antimicrobial susceptibility testing could only be performed on patients who were culture-positive for *H. pylori*, because antimicrobial susceptibility testing requires viable cultured isolates. Therefore, among the enrolled patients, only those with positive cultures were included in the resistance analyses. The remaining patients, although *H. pylori*-positive by other diagnostic methods, were culture-negative and were excluded from resistance-related analyses.

### 2.3 Study procedures

**Figure 1:**
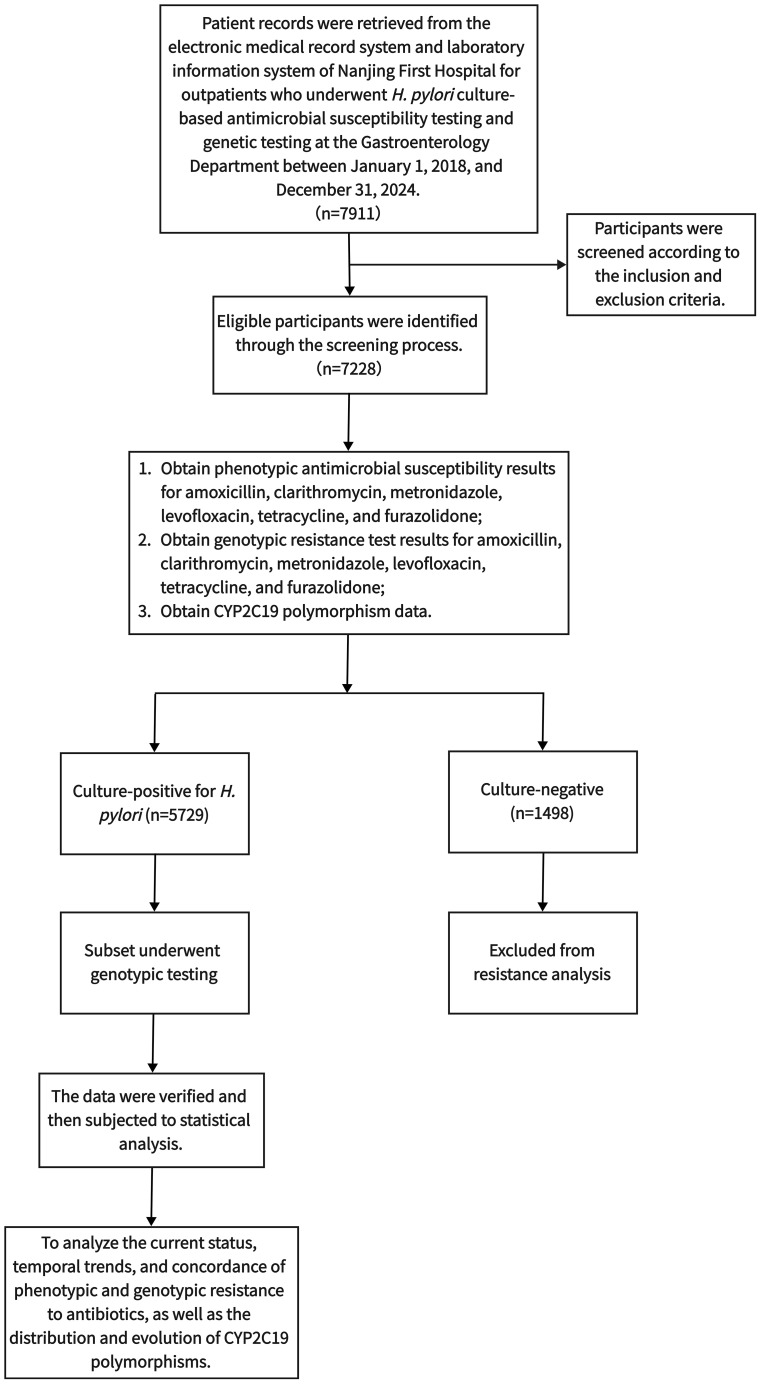
Study flowchart. †: *H. pylori*, *Helicobacter pylori*.

Researchers retrieved and screened records of outpatients who underwent *H. pylori* culture-based phenotypic resistance testing and genetic testing at the Gastroenterology Department of our hospital between January 1, 2018, and December 31, 2024, strictly following the pre-defined inclusion and exclusion criteria. Data from eligible cases were extracted, including demographic information, phenotypic susceptibility results, resistance gene test results, and CYP2C19 polymorphism data. After data cleaning and verification, all data were uniformly supervised and managed by the Gastroenterology Department of Nanjing First Hospital. Statistical analyses were then performed to assess the phenotypic and genotypic resistance status, trends, and concordance for the relevant antibiotics, as well as the distribution and evolution of CYP2C19 polymorphisms. The overall study technical roadmap is shown in **Figure 1**.

### 2.4 Methods

All *H. pylori* culture and antimicrobial susceptibility testing data as well as molecular biology data used in this analysis were obtained from routine laboratory results stored in the hospital information system. Following medical record screening and data extraction, the procedures for *H. pylori* culture, susceptibility testing, and molecular analysis were retrospectively reviewed and are summarized below.

#### 2.4.1 Sample collection

Gastric mucosal samples were collected from patients during gastroscopy. For each patient, the sample consisted of two biopsy specimens taken from the gastric antrum and one biopsy specimen taken from the gastric body. All three specimens were placed together in a single sterile collection tube. From this pooled specimen tube, the samples were allocated for parallel testing as follows: one portion was used for *H. pylori* culture and subsequent phenotypic antimicrobial susceptibility testing; another portion was used for DNA extraction. Thus, the same specimen tube served as the source for all experiments, ensuring that culture, phenotypic antimicrobial susceptibility testing, genotypic resistance testing, and CYP2C19 genotyping were all performed on biological material derived from the same set of biopsies.

#### 2.4.2 *H. pylori* culture

*H. pylori* was cultured on Columbia agar plates supplemented with 5% sheep blood. The agar contained vancomycin (1 mg/ml), polymyxin B (0.5 mg/ml), and amphotericin B (0.5 mg/ml) to suppress contaminating microorganisms. Plates were incubated in a microaerobic atmosphere (10% CO_2_, 5% O_2_) at 37 °C for 96–120 hours. Following culture, the obtained isolates or colonies underwent essential confirmatory testing.

#### 2.4.3 Phenotypic resistance testing

Phenotypic antimicrobial susceptibility testing was performed only on culture-positive *H. pylori* isolates. Phenotypic resistance of the strains was determined using the Kirby–Bauer disk diffusion method. Antibiotic disks used in the assay included clarithromycin (15 mg), levofloxacin (5 mg), amoxicillin (10 mg), furazolidone (100 mg), tetracycline (30 mg), and metronidazole (5 mg). A bacterial suspension adjusted to a 0.5 McFarland standard was evenly spread on the culture plate. Antibiotic disks were then gently placed onto the surface of the solidified agar medium. The diameter of the inhibition zone was measured to evaluate the resistance level of *H. pylori* to each antibiotic. The interpretive criteria for classifying isolates as resistant, intermediate, or susceptible are presented in **Supplementary Table 1**, based on previous reports (Mégraud et al., 1999; Zhong et al., 2021). Quality control was performed using the reference strain *H. pylori* ATCC43504, which is recommended by CLSI document for antimicrobial susceptibility testing of *H. pylori*. According to CLSI guidelines, susceptibility testing of ATCC43504 was conducted concurrently with the test isolates. The reliability of the test results was confirmed only when the susceptibility profile of ATCC43504 fell within the specified acceptable range and exhibited expected sensitivity (CLSI, 2015).

#### 2.4.4 Genotypic resistance testing for *H. pylori*

All molecular analyses were performed using DNA extracted from the same aliquot of the pooled biopsy specimens.

##### 2.4.4.1 DNA extraction from samples

In this study, the collected gastric mucosal specimens were immediately immersed in liquid transport medium for preservation. DNA was subsequently extracted from the sample-containing transport medium using the HiPure bacterial DNA kit (Magen Biotech, Guangzhou, China).

##### 2.4.4.2 Performing PCR amplification

Multi-PCR was performed to amplify the specific fragments of target antibiotic resistance-associated genes, including *gyrA*, *23S rRNA*, *PBP1A*, *porD*, *rdxA*, and *16S rRNA*. The selection of these loci was based on established literature and widely used molecular diagnostics. Specifically, for clarithromycin, mutations in *23S rRNA* are well-established as the primary resistance determinants (Jiang et al., 2022). For levofloxacin, mutations at codons 87 and 91 in *gyrA* are the most prevalent in China and globally (Zhong et al., 2021). For amoxicillin, tetracycline, furazolidone, and metronidazole, we selected the most commonly reported loci in Chinese populations (*PBP1A* for amoxicillin; *16S rRNA* for tetracycline; *porD* for furazolidone; *rdxA* for metronidazole) (Zhong et al., 2021), while acknowledging that resistance mechanisms for these drugs are less straightforward and may involve multiple genes or non-mutation mechanisms. Based on the sequence of reference *H. pylori* strain 26695 available on the GenBank website, primers were designed to detect point mutations which were listed in **Table 1**.

**Table 1:** Primer sequences for antibiotic resistance-associated mutations detection of the *H. pylori*.

Gene	Loci of interpretation	PCR amplification primers (5’–3’)	Significance
*23SrRNA*	A2142C/G	Forward: ACGTTGGATGTGAAAATTCCTCCTACCCGC Reverse: ACGTTGGATGATCCTGCGCATGATATTCCC	Clarithromycin resistance
	A2143C/G		
*gyrA*	G271T(D91Y)	Forward: ACGTTGGATGGCATGGAAAAATCTTGCGCC Reverse: ACGTTGGATGGATTGGTAAATACCACCCCC	Levofloxacin resistance
	G271A(D91N)		
	A272G(D91G)		
	A272T(D91V)		
	T/C261A/T(N87K)	Forward: ACGTTGGATGGATTGGTAAATACCACCCCC Reverse: ACGTTGGATGGCATGGAAAAATCTTGCGCC	
*PBP1A*	C1242A/G(S414R)	Forward: ACGTTGGATGAACAGCGAACAAAACCACGC Reverse: ACGTTGGATGTCTTGCAAGGTTACAAGCCC	Amoxicillin resistance
	C1667G(T556S)	Forward: ACGTTGGATGAAATGGCACAGGGAGTTTGG Reverse: ACGTTGGATGTAAAGCCAATGAACCAAGCG	
*porD*	G343A(A115T)	Forward: ACGTTGGATGCGCTATGGATGTTTGAAGAAC Reverse: ACGTTGGATGCCATCCCACACTTCTATCTC	Furazolidone resistance
	C347T(T116A)		
	A346G(T116A)		
*rdxA*	G565T	Forward: ACGTTGGATGATCAATAAGCCTAAAATCGCATG Reverse: ACGTTGGATGGAAAACACCCCTAAAAGAGCG	Metronidazole resistance
	G616A		
*16SrRNA*	AGA926-928TTC	Forward: ACGTTGGATGAACTCAAAGGAATAGACGGG Reverse: ACGTTGGATGGTCAAGCCTAGGTAAGGTTC	Tetracycline resistance

##### 2.4.4.3 Performing shrimp alkaline phosphatase treatment

PCR products (5 μL) were added with 10×SAP Buffer 0.17 μL, shrimp alkaline phosphatase (SAP) Enzyme (1.7 U/μL) 0.3 μL, and ddH_2_O 1.53 μL to a final volume of 7 μL. The SAP treatment condition was set as: 37 °C 40 min, and 85 °C 5 min by using the ETC811 PCR amplification instrument (Eastwin Scientific Equipment Inc., Suzhou, Jiangsu, China).

##### 2.4.4.4 Performing the extension reaction

Extend primers were designed for performing the single base extension, as listed in **Supplementary Table 1**. The reaction conditions were as follows: re-denaturation at 95 °C for 30 s, followed by 40 cycles of denaturation at 95 °C for 5 s, 5 cycles of annealing at 52 °C for 5 s and extension at 80 °C for 5 s, and final elongation at 72 °C for 3 min.

##### 2.4.4.5 Mass spectrum acquisition by MALDI-TOF MS

The extension product was detected using MALDI-TOF MS for molecular weight detection (Agena Bioscience, San Diego, California, USA). Based on the molecular weight labeling, the specific point of antibiotic resistance-associated mutations occurred in the sample could be determined by using the MassARRAY^®^Typer 4.1 software.

##### 2.4.4.6 CYP2C19 genotyping

CYP2C19 genotyping was performed using the same DNA template extracted from the gastric mucosal biopsy specimens. CYP2C19 polymorphisms were detected using PCR amplification combined with agarose gel electrophoresis. Bidirectional sequencing was performed on a 3500DX Genetic Analyzer, and the resulting sequencing chromatograms were analyzed using Chromas software to identify common CYP2C19 polymorphic sites. Genotypes and metabolic phenotypes were then determined based on the sequence results. We define metabolic phenotypes as: Extensive metabolizer (EM): ∗1/∗1; Intermediate metabolizer (IM): ∗1/∗2, ∗1/∗3,∗2/∗17 or ∗3/∗17; Poor metabolizer (PM): ∗2/∗2,∗2/∗3 or ∗3/∗3; Ultrametabolizer (UM):∗17/∗17 or ∗1/∗17.

### 2.5 Statistical analysis

**Figure 2:**
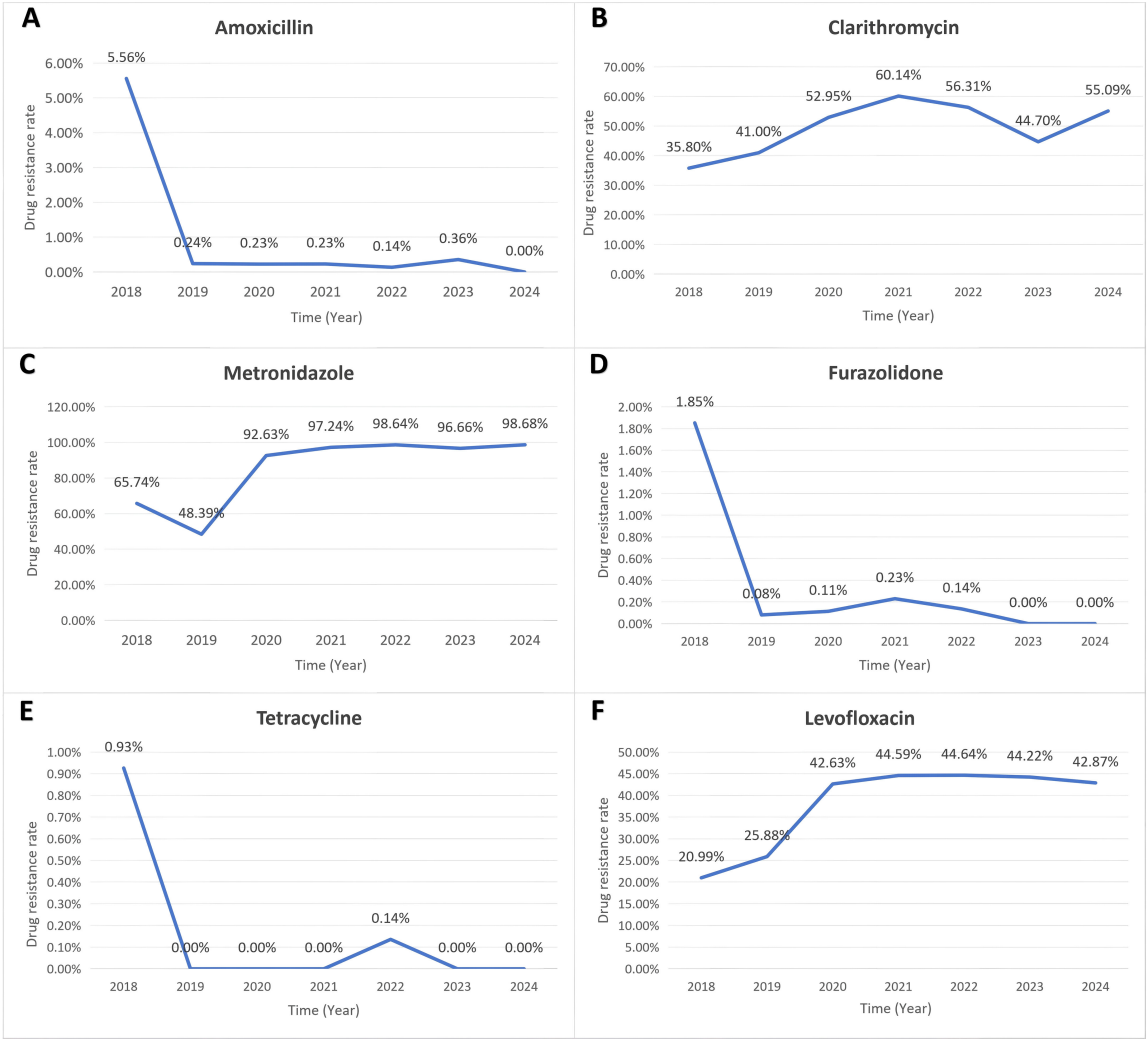
Temporal trends in resistance rates for six antibiotics (2018–2024). **(A)** Amoxicillin; **(B)** Clarithromycin; **(C)** Metronidazole; **(D)** Furazolidone; **(E)** Tetracycline; **(F)** Levofloxacin.

Statistical analyses were performed using SPSS software (version 27.0). All tests were two-sided, and a P-value < 0.05 was considered statistically significant. Continuous data are presented as mean ± standard deviation (SD), and categorical data are presented as frequencies and percentages. For all resistance rates, exact binomial 95% confidence intervals (CIs) were calculated using the Clopper-Pearson method. The temporal trends in annual phenotypic resistance rates from 2018 to 2024 shown in **Figure 2** are presented descriptively; we described the observed changes in resistance rates over time without performing formal statistical trend tests, as the primary aim of this analysis was to provide an overview of resistance dynamics rather than to test for statistically significant trends, and this descriptive approach is appropriate given the exploratory nature of this analysis and the varying sample sizes across years. Given the retrospective nature of this study, missing data were not imputed, and for each analysis, the denominator reflects the actual number of patients or isolates with available results for the specific test, as explicitly stated in the results section for each analysis. Of note, at our institution, each patient undergoes *H. pylori* culture and antimicrobial susceptibility testing only once during the study period, and we have carefully reviewed and verified the data to ensure that no duplicate patients were included. The Kappa consistency test was used to evaluate the concordance between phenotypic and genotypic resistance results. The Kappa coefficient ranges from -1 to 1. A Kappa value greater than 0.75 generally indicates excellent agreement between the two methods. The closer the Kappa value is to 1, the higher the level of agreement between the two methods in determining antimicrobial resistance. Sensitivity, specificity, positive predictive value, and negative predictive value were also calculated for genotypic testing using phenotypic antimicrobial susceptibility testing as the reference standard. To identify patient-level predictors of antibiotic resistance, multivariate binary logistic regression analysis was performed with resistance status as the dependent variable and year, age, and sex as independent variables. Year was entered as a continuous variable centered at 2021. Age was entered as a continuous variable using the midpoints of each age group (<40 years = 25, 40–60 years = 50, >60 years = 70). Sex was entered as a categorical variable with female as the reference group. Results are presented as odds ratios (ORs) with 95% confidence intervals (CIs). For amoxicillin, tetracycline, and furazolidone, multivariate analysis was not performed due to the extremely low number of resistant cases (7, 0, and 1 respectively across all years), which would render the model unstable.

## 3 Results

### 3.1 Baseline characteristics of the study population

After screening according to the pre-specified inclusion and exclusion criteria, a total of 7,227 participants were enrolled in this study. Among them, 3,649 (50.49%) were male and 3,578 (49.51%) were female. The mean age was 47.93 ± 13.42 years (range: 11 to 91 years).

### 3.2 Antimicrobial resistance of *H. pylori*

#### 3.2.1 Overall resistance profile

Among the 7227 enrolled participants who met the inclusion criteria, 5729 (79.27%) were culture-positive for *H. pylori* and successfully underwent phenotypic antimicrobial susceptibility testing. The remaining 1498 participants (20.73%), although *H. pylori*-positive by other diagnostic methods (¹³C-urea breath test, rapid urease test, or histopathological examination), were culture-negative and were therefore excluded from all resistance analyses. All subsequent resistance data reported in this section are based on the 5729 culture-positive isolates. Overall, the phenotypic resistance rates varied considerably among the six commonly used antibiotics. Metronidazole had the highest resistance rate at 84.45% (4838/5729, 95% CI: 83.50–85.37), indicating a high level of resistance. Clarithromycin and levofloxacin followed, with resistance rates of 50.01% (2865/5729, 95% CI: 48.71–51.31) and 38.59% (2211/5729, 95% CI: 37.33–39.87), respectively. Amoxicillin showed a low resistance rate of 0.51% (29/5729, 95% CI: 0.34–0.73). Furazolidone and tetracycline had very low resistance rates of 0.19% (11/5729, 95% CI: 0.10–0.34) and 0.07% (4/5729, 95% CI: 0.02–0.18), respectively. These findings indicate a severe resistance situation in the Nanjing region for metronidazole, clarithromycin, and levofloxacin, while *H. pylori* remains highly susceptible to amoxicillin, furazolidone, and tetracycline.

#### 3.2.2 Temporal trends in resistance rates for six antibiotics (2018–2024)

Between 2018 and 2024, the annual phenotypic resistance rates of the six commonly used antibiotics showed distinct temporal trends, as shown in **Figure 2**. The resistance rates for amoxicillin, furazolidone, and tetracycline remained consistently very low. The amoxicillin resistance rate declined rapidly from 5.56% (18/324) in 2018 to 0.24% (3/1244) in 2019, and thereafter fluctuated between 0.00% and 0.36%, with no resistant strains detected in 2024. The furazolidone resistance rate decreased annually from 1.85% (6/324) in 2018, and no resistance was detected for two consecutive years starting in 2023. For tetracycline, resistance was sporadically observed only in 2018 (0.93%, 3/324) and 2022 (0.14%, 1/737), with no resistance detected in all other years. In contrast, the resistance situation for metronidazole, clarithromycin, and levofloxacin was much more severe. After a brief decline to 48.39% in 2019, the metronidazole resistance rate rapidly increased and remained above 92% since 2020, ranging from 96.66% to 98.68% between 2021 and 2024, peaking at 98.68% in 2024. The clarithromycin resistance rate generally showed a fluctuating upward trend, rising from 35.80% in 2018 to 60.14% in 2021, followed by a slight decline, though it remained as high as 55.09% in 2024. The levofloxacin resistance rate increased steadily from 20.99% in 2018, reaching 44.59% in 2021, and has since remained stable.

#### 3.2.3 Distribution of *H. pylori* resistance profiles

**Figure 3:**
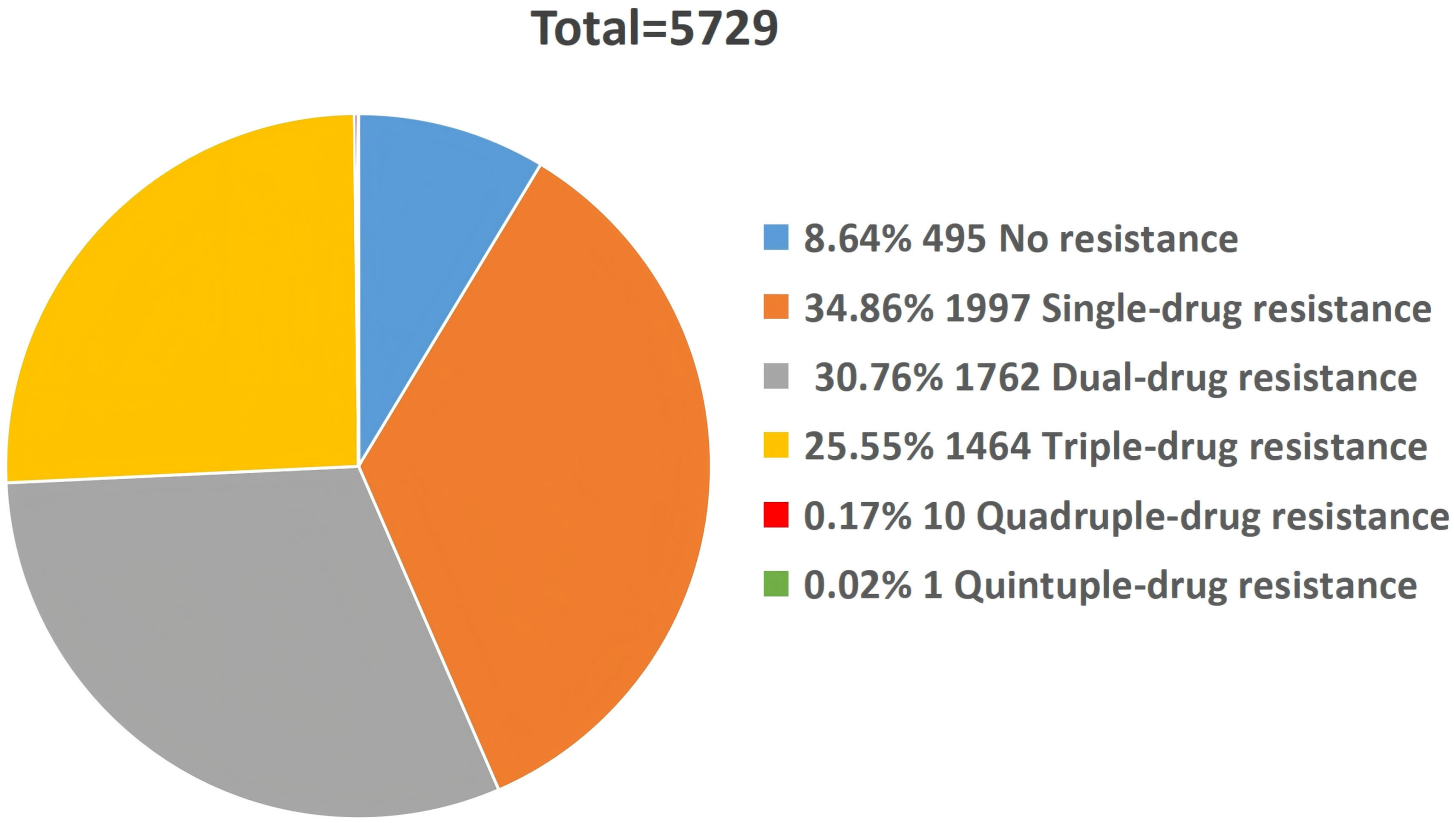
Distribution of resistance profiles among *H. pylori* strains (n=5729).

As shown in **Table 2** and **Figure 3**, the most common resistance pattern was single-drug resistance (34.86% of all strains), predominantly metronidazole alone (29.76%). Dual-drug resistance accounted for 30.76% of strains, with the combination of clarithromycin and metronidazole being the most frequent (18.76%), followed by metronidazole and levofloxacin (9.95%). Multidrug resistance (resistance to three or more drugs) was observed in 25.75% of strains, with the triple combination of clarithromycin, metronidazole, and levofloxacin accounting for the vast majority (25.33%). Pan-susceptible strains comprised only 8.64% of the cohort.

**Table 2:** Distribution of resistance patterns among *H. pylori* strains.

Resistance pattern	Number of strains	Percentage of all strains (%)	Percentage of resistant strains (%)
Pan-susceptible	495	8.64 (495/5729)	-
Single-drug resistance	1997	34.86 (1997/5729)	38.16 (1997/5234)
Amoxicillin only	2	0.03 (2/5729)	0.04 (2/5234)
Clarithromycin only	213	3.72 (213/5729)	4.07 (213/5234)
Metronidazole only	1705	29.76 (1705/5729)	32.58 (1705/5234)
Levofloxacin only	77	1.34 (77/5729)	1.47 (77/5234)
Dual-drug resistance	1762	30.76 (1762/5729)	33.67 (1762/5234)
Clarithromycin + Metronidazole	1075	18.76 (1075/5729)	20.54 (1075/5234)
Clarithromycin + Levofloxacin	103	1.80 (103/5729)	1.97 (103/5234)
Metronidazole + Levofloxacin	570	9.95 (570/5729)	10.89 (570/5234)
Other dual-resistance combinations*	14	0.24 (14/5729)	0.27 (14/5234)
Multidrug resistance (≥3 drugs)	1475	25.75 (1475/5729)	28.17 (1475/5234)
Clarithromycin + Metronidazole + Levofloxacin	1451	25.33 (1451/5729)	27.72 (1451/5234)
Other multidrug-resistant patterns†	24	0.42 (24/5729)	0.45 (24/5234)
Total resistant strains	5,234	91.36 (5234/5729)	100.00 (5234/5234)
Total strains	5,729	100.00 (5729/5729)	-

*Other dual-resistance combinations include: amoxicillin + metronidazole (n=9), clarithromycin + furazolidone (n=1), metronidazole + furazolidone (n=4).

†Other multidrug-resistant patterns include various combinations of three or more drugs, each with fewer than 10 cases.

### 3.3 CYP2C19 genotype distribution

CYP2C19 genotyping was performed in 1,654 participants. As shown in **Table 3**, among all tested individuals, 726 (43.89%) were extensive metabolizers, all with the *1/*1 genotype; 732 (44.26%) were intermediate metabolizers, including 649 with the *1/*2 genotype, 82 with the *1/*3 genotype, and 1 with the *2/*17 genotype; 193 (11.67%) were poor metabolizers, including 161 with the *2/*2 genotype, 30 with the *2/*3 genotype, and 2 with the *3/*3 genotype; and 3 (0.18%) were ultrametabolizers, all with the *1/*17 genotype. In summary, in the Nanjing population, the extensive and intermediate metabolizer phenotypes were similarly prevalent, while the poor metabolizer phenotype was less common and the ultrametabolizer phenotype was rare.

**Table 3:** Distribution of CYP2C19 genotypes and metabolic phenotypes.

Metabolic phenotype	Genotype	Number of cases	Percentage (%)
Ultrametabolizers	*1/*17	3	0.18
Extensive metabolizers	*1/*1	726	43.89
Intermediate metabolizers	*1/*2	649	39.24
	*1/*3	82	4.96
	*2/*17	1	0.06
Poor metabolizers	*2/*2	161	9.73
	*2/*3	30	1.81
	*3/*3	2	0.12
Total		1654	100.00

Note: The ‘*’ is the international nomenclature symbol used to designate distinct allelic variants of the CYP2C19 gene. The number denotes a specific mutation type. Results are reported as diplotype combinations to predict drug metabolism phenotypes.

### 3.4 Concordance analysis between phenotypic and genotypic resistance for six antibiotics

Of the 5729 culture-positive patients, a subset underwent genotypic testing. Using phenotypic antimicrobial susceptibility testing as the reference standard, we evaluated the diagnostic performance of genotypic testing for resistance to six commonly used antibiotics and assessed the concordance between the two methods. Isolates with intermediate susceptibility according to the criteria in **Supplementary Table 1** were excluded from the concordance analysis. The numbers of samples tested for each antibiotic by genotypic testing were as follows: 5,413 for clarithromycin, 1,333 for levofloxacin, 1,347 for amoxicillin, 1,345 for tetracycline, 1,366 for furazolidone, and 1,316 for metronidazole. Detailed results are presented in **Tables 4** and **5**.

**Table 4:** Comparison of phenotypic and genotypic resistance testing results for six antibiotics.

Antibiotic	Test type	Genotypic resistant (Mutant)	Genotypic susceptible (Wild-Type)	Total	Sensitivity (%) (95% CI)	Specificity (%) (95% CI)	PPV (%) (95% CI)	NPV (%) (95% CI)	Overall agreement (%) (95% CI)
Clarithromycin (n=5413)	Phenotypic Resistant	2765	69	2834	97.57 (96.95–98.09)	92.71 (91.61–93.73)	93.63 (92.73–94.45)	97.20 (96.45–97.84)	95.25
	Phenotypic Susceptible	188	2391	2579					(94.65–95.80)
	Total	2953	2460	5413					
Levofloxacin(n=1333)	Phenotypic Resistant	775	27	802	96.63 (95.22–97.78)	95.29 (93.19–97.00)	96.88 (95.48–97.97)	94.93 (92.85–96.61)	96.10
	Phenotypic Susceptible	25	506	531					(94.92–97.11)
	Total	800	533	1333					
Amoxicillin (n=1347)	Phenotypic Resistant	6	1	7	85.71 (42.13–99.64)	74.63 (72.26–76.91)	1.73 (0.71–3.55)	99.90 (99.39–99.99)	74.69
	Phenotypic Susceptible	340	1000	1340					(72.28–77.01)
	Total	346	1001	1347					
Tetracycline (n=1345)	Phenotypic Resistant	0	0	0	/	85.65 (83.67–87.47)	/	100.00 (99.66–100.00)	85.65
	Phenotypic Susceptible	193	1152	1345					(83.67–87.47)
	Total	193	1152	1345					
Furazolidone(n=1366)	Phenotypic Resistant	1	0	1	100.00 (2.50–100.00)	23.81 (21.59–26.14)	0.10 (0.00–0.53)	100.00 (98.84–100.00)	23.86
	Phenotypic Susceptible	1040	325	1365					(21.64–26.19)
	Total	1041	325	1366					
Metronidazole (n=1316)	Phenotypic Resistant	1281	5	1286	99.61 (99.07–99.88)	6.67 (0.82–22.07)	97.86 (96.94–98.58)	28.57 (4.22–70.96)	97.49
	Phenotypic Susceptible	28	2	30					(96.50–98.32)
	Total	1309	7	1316					

PPV, positive predictive value; NPV, negative predictive value. Isolates with intermediate susceptibility were excluded from this analysis. See **Table 5** for Kappa concordance and corresponding P-values.

**Table 5:** Concordance evaluation of two detection methods for *H. pylori* resistance.

Antibiotic	Value	95% CI	Asymp. Std. Error	Approx. Tb	Approx. Sig.
Clarithromycin	0.90	0.89–0.92	0.01	155.71	<0.001
Levofloxacin	0.92	0.90–0.94	0.01	83.07	<0.001
Amoxicillin*	0.02	-	0.05	0.52	0.60
Tetracycline*	0.00	-	0.07	0.00	1.00
Furazolidone*	0.00	-	0.02	0.03	0.98
Metronidazole	0.10	-0.20–0.40	0.15	0.65	0.52

*For amoxicillin, tetracycline, and furazolidone, the number of phenotypically resistant isolates was extremely low 7, 0, and 1 respectively. The Kappa coefficient is statistically unstable when the number of positive cases is very small or zero. Therefore, 95% CIs are not reported for these antibiotics, and these low Kappa values should not be interpreted as evidence of poor concordance. Note: See **Table 4** for detailed diagnostic performance metrics including sensitivity, specificity, PPV, and NPV. Isolates with intermediate susceptibility were excluded from this analysis.

For clarithromycin, genotypic testing demonstrated excellent diagnostic performance with a sensitivity of 97.57% (95% CI: 96.95–98.09), specificity of 92.71% (95% CI: 91.61–93.73), PPV of 93.63% (95% CI: 92.73–94.45), NPV of 97.20% (95% CI: 96.45–97.84), accuracy of 95.25% (95% CI: 94.65–95.80), and a Kappa value of 0.9046 (95% CI: 0.8921–0.9171, P<0.001). For levofloxacin, genotypic testing also showed excellent performance with a sensitivity of 96.63% (95% CI: 95.22–97.78), specificity of 95.29% (95% CI: 93.19–97.00), PPV of 96.88% (95% CI: 95.48–97.97), NPV of 94.93% (95% CI: 92.85–96.61), accuracy of 96.10% (95% CI: 94.92–97.11), and a Kappa value of 0.9187 (95% CI: 0.9024–0.9350, P<0.001).

For amoxicillin, tetracycline, and furazolidone, the number of phenotypically resistant isolates was extremely low (7, 0, and 1 respectively). Due to this extreme imbalance, the Kappa coefficient is statistically unstable and may be misleading. Therefore, we emphasize the predictive values rather than Kappa for these antibiotics. The negative predictive values were very high for all three: amoxicillin 99.90% (95% CI: 99.39–99.99), tetracycline 100.00% (95% CI: 99.66–100.00), and furazolidone 100.00% (95% CI: 98.84–100.00), indicating that a negative genotypic test reliably rules out resistance. However, the positive predictive values were very low: amoxicillin 1.73% (95% CI: 0.71–3.55), tetracycline not applicable, and furazolidone 0.10% (95% CI: 0.00–0.53), reflecting the low prevalence of phenotypic resistance and the high number of false-positive genotypic results. These low Kappa values (0.0239, 0, and 0.0005) should not be interpreted as evidence of poor concordance; they simply reflect the statistical limitation imposed by the rarity of phenotypic resistance. For metronidazole, genotypic testing showed high sensitivity of 99.61% (95% CI: 99.07–99.88) and high PPV of 97.86% (95% CI: 96.94–98.58), but very low specificity of 6.67% (95% CI: 0.82–22.07) and low NPV of 28.57% (95% CI: 4.22–70.96), with a Kappa value of 0.1004 (95% CI: -0.1998–0.4006, P = 0.52).

### 3.5 Analysis of resistance-associated gene mutations for clarithromycin and levofloxacin

This study further analyzed the resistance-associated genes *23S rRNA* and *gyrA* for clarithromycin and levofloxacin, respectively, both of which showed excellent concordance. The results of the mutation analysis are presented in **Table 6**. It should be noted that this analysis included all samples that underwent genetic testing, including those with intermediate susceptibility. Therefore, the number of cases tested differs from that in the concordance analysis described above. Among the 3,177 samples that completed *23S rRNA* gene testing, the A2143G mutation was the predominant type, detected in 3,143 samples (98.93%, 3,143/3,177), of which 2,786 were homozygous mutations (87.69%, 2,786/3,177) and 357 were heterozygous mutations (11.24%, 357/3,177). The A2142G mutation was detected in 30 samples (0.95%, 30/3,177). One sample (0.03%, 1/3,177) carried a homozygous A2143C mutation. Among the 826 samples that completed *gyrA* gene testing, the N87K single mutation was the most common, detected in 434 samples (52.54%, 434/826), followed by D91N (14.16%, 117/826), D91G (10.17%, 84/826), N87I (7.26%, 60/826), and N91Y (6.78%, 56/826). Compound mutations were most frequently N87K combined with D91G (1.69%, 14/826) and N87K combined with D91N (1.45%, 12/826), with all other compound mutation types occurring at frequencies below 1%.

**Table 6:** Distribution of resistance-associated gene mutations for clarithromycin and levofloxacin.

Mutated gene	Mutation site	Number	Mutation frequency (%)
*23S rRNA* (n=3177)	A2142G homozygous mutation	26	0.82
	A2142G heterozygous mutation	4	0.13
	A2143G homozygous mutation	2786	87.69
	A2143G heterozygous mutation	357	11.24
	A2142G heterozygous combined with A2143G heterozygous mutation	3	0.09
	A2143C homozygous mutation	1	0.03
*gyrA* (n=826)	N87K	434	52.54
	D91N	117	14.16
	D91G	84	10.17
	N87I	60	7.26
	N91Y	56	6.78
	D87Y	11	1.33
	N87R	2	0.24
	N87K, D91G	14	1.69
	N87K, D91N	12	1.45
	N87K, D91Y	6	0.73
	D91N, D91G	5	0.61
	N91Y, D91G	4	0.48
	N87I, N87K	5	0.61
	N91Y, D91N	4	0.48
	N87I, D91N	3	0.36
	N87I, N91Y	2	0.24
	N87I, D91G	3	0.36
	D87Y, D91N	1	0.12
	N87K, D87Y	2	0.24
	D91G, D91N, N91Y	1	0.12

### 3.6 Subgroup analysis of pediatric/adolescent patients (age <no><18</no> years)

Among the 7,227 enrolled patients, 26 patients (0.36%) were under 18 years of age. Of these 26 patients, 21 (80.77%) were culture-positive for *H. pylori* and underwent phenotypic antimicrobial susceptibility testing. Amoxicillin, furazolidone, and tetracycline all showed 0% resistance, with all 21 isolates being susceptible. The resistance rate to clarithromycin was 76.19% (16/21), to metronidazole was 80.95% (17/21), and to levofloxacin was 19.05% (4/21). The resistance patterns in pediatric patients were generally consistent with those observed in the overall cohort, although the small sample size precludes robust statistical comparison between age groups.

CYP2C19 genotyping was performed in 13 of the 26 pediatric patients (50.0%). The distribution of metabolic phenotypes was as follows: extensive metabolizers in 6 patients (46.15%), intermediate metabolizers in 3 patients (23.08%), poor metabolizers in 4 patients (30.77%), and no ultrametabolizers were identified. Due to the limited sample size, formal statistical comparison with adult patients was not performed.

### 3.7 Multivariate analysis of factors associated with antibiotic resistance

To identify patient-level predictors of resistance, multivariate binary logistic regression analysis was performed with year, age, and sex as independent variables. For amoxicillin, tetracycline, and furazolidone, multivariate analysis was not performed due to the extremely low number of resistant cases across all years (7, 0, and 1 respectively). The results for clarithromycin, levofloxacin, and metronidazole are presented in **Table 7**.

**Table 7:** Multivariate logistic regression analysis of factors associated with *H. pylori* antibiotic resistance.

Antibiotic	Variable	OR	95% CI	P-value
Clarithromycin	Year	1.096	1.061–1.132	<0.001
	Age	0.994	0.960–1.029	0.738
	Sex	1.038	0.947–1.138	0.432
Levofloxacin	Year	1.132	1.095–1.170	<0.001
	Age	0.989	0.954–1.025	0.533
	Sex	1.042	0.947–1.147	0.410
Metronidazole	Year	1.184	1.128–1.243	<0.001
	Age	0.987	0.940–1.036	0.580
	Sex	0.958	0.841–1.092	0.516

For amoxicillin, tetracycline, and furazolidone, multivariate analysis was not performed due to the extremely low number of resistant cases across all years (7, 0, and 1 respectively). OR, odds ratio; CI, confidence interval.

As shown in **Table 7**, year was a significant predictor of resistance for all three antibiotics. For clarithromycin, the odds ratio per one-year increase was 1.096 (95% CI: 1.061–1.132, P < 0.001); for levofloxacin, OR = 1.132 (95% CI: 1.095–1.170, P < 0.001); and for metronidazole, OR = 1.184 (95% CI: 1.128–1.243, P < 0.001). These findings confirm the temporal increasing trends observed in our descriptive analysis (**Figure 2**). In contrast, age was not a significant predictor for any of the three antibiotics. The odds ratios per 10-year increase were 0.994 (95% CI: 0.960–1.029, P = 0.738) for clarithromycin, 0.989 (95% CI: 0.954–1.025, P = 0.533) for levofloxacin, and 0.987 (95% CI: 0.940–1.036, P = 0.580) for metronidazole, indicating that age does not substantially influence resistance risk in this cohort. Similarly, sex was not a significant predictor for any of the three antibiotics. Compared with female patients, male patients showed no significant difference in resistance risk for clarithromycin (OR = 1.038, 95% CI: 0.947–1.138, P = 0.432), levofloxacin (OR = 1.042, 95% CI: 0.947–1.147, P = 0.410), or metronidazole (OR = 0.958, 95% CI: 0.841–1.092, P = 0.516). 

## 4 Discussion

The continued rise in antibiotic resistance rates of *H. pylori* has become a global public health concern, with resistance patterns varying significantly across different regions. This study, based on antimicrobial susceptibility data and genetic testing results from *H. pylori* culture-positive patients in the Nanjing region between 2018 and 2024, systematically analyzed the phenotypic and genotypic resistance profiles, annual trends, phenotype-genotype concordance, CYP2C19 polymorphisms, and resistance-associated gene mutations for six commonly used antibiotics. These findings provide important reference evidence to guide individualized treatment of *H. pylori* infection in this region.

Our study found significant differences in the resistance rates of *H. pylori* to six antibiotics in the Nanjing region. The resistance rate to metronidazole was as high as 84.45%, followed by clarithromycin (50.01%) and levofloxacin (38.59%). In contrast, amoxicillin, furazolidone, and tetracycline remained highly susceptible, with resistance rates all below 0.6%. These findings are generally consistent with previous studies from this region (Jiang et al., 2022; Wei et al., 2023). Annual trend analysis showed that the metronidazole resistance rate remained above 92% since 2020, reaching a peak of 98.68% in 2024, indicating a persistently high level. The clarithromycin resistance rate fluctuated but generally increased from 35.80% in 2018 to 60.14% in 2021, showing an overall upward trend. The levofloxacin resistance rate rose from 20.99% in 2018 to 44.59% in 2021 and then stabilized. These trends may be related to the widespread clinical use of clarithromycin, levofloxacin, and metronidazole without adherence to antimicrobial stewardship principles. In recent years, the widespread use of empirical eradication therapy without considering bacterial resistance has led to a rapid and significant increase in antibiotic resistance worldwide, becoming a major cause of *H. pylori* treatment failure and declining eradication rates (Tshibangu-Kabamba and Yamaoka, 2021). As Nanjing is a region with high resistance to clarithromycin, metronidazole, and levofloxacin, careful selection of antibiotics in eradication regimens is particularly important. In our study, amoxicillin, furazolidone, and tetracycline consistently maintained very low resistance levels, suggesting that these drugs remain reliable options for *H. pylori* eradication in this region. Among them, amoxicillin, a β-lactam antibiotic, offers advantages such as strong bactericidal activity, high safety, and a low propensity for resistance, making it particularly valuable in clinical practice. In recent years, dual therapy containing amoxicillin and an acid suppressant has been shown to be effective in multiple clinical studies with a low incidence of adverse events (Zhou et al., 2024; Hu et al., 2025). The latest Chinese guidelines also state that high-dose dual therapy, like bismuth-containing quadruple therapy, can be used for both first-line and second-line *H. pylori* eradication (Zhou et al., 2022). Given these findings and the high susceptibility of *H. pylori* to amoxicillin in this region, we strongly recommend amoxicillin-based high-dose dual therapy as a first-line empiric strategy. Alternatively, bismuth-containing quadruple regimens that substitute amoxicillin, furazolidone, or tetracycline for the highly resistant antibiotics are also highly effective. Additionally, we advocate for the adoption of amoxicillin-centered empiric regimens as the standard of care in the Nanjing region, reserving metronidazole, clarithromycin, and levofloxacin for susceptibility-confirmed cases only. This approach is of great clinical value and public health significance, as it directly addresses the growing challenge of antibiotic resistance while maintaining treatment efficacy.

The resistance profile in this study showed that among the 5,729 culture-positive strains, only 8.64% were pan-susceptible, while the proportions of single-drug resistant, dual-resistant, and multidrug-resistant strains varied markedly. Metronidazole had the highest resistance rate and accounted for the largest proportion of single-drug resistant strains. Co-resistance to clarithromycin and levofloxacin was common among dual-resistant and multidrug-resistant strains, suggesting a relatively high prevalence of cross-resistance in this region. This finding is generally consistent with reports from other regions in China (Zhong et al., 2021) and may be related to the extensive clinical use of clarithromycin and levofloxacin. The high detection rate of multidrug-resistant strains further complicates eradication therapy and limits the choice of empirical treatment regimens. Therefore, in the management of *H. pylori* infection, dynamic surveillance of antimicrobial resistance should be strengthened, and individualized treatment strategies should be developed based on resistance profiles to address the growing challenge of resistance.

CYP2C19 polymorphism is a key genetic factor influencing the metabolic activity of proton pump inhibitors, thereby affecting the efficacy of PPI-based *H. pylori* eradication regimens. In this study, CYP2C19 genotyping was performed specifically to inform PPI dosing and selection for individualized acid suppression therapy. The results showed that extensive metabolizers accounted for 43.89%, intermediate metabolizers for 44.26%, poor metabolizers for 11.67%, and ultrametabolizers for only 0.18%. The combined proportion of extensive and intermediate metabolizers reached 88.33%, indicating that the majority of the population in this region has a strong capacity to metabolize PPIs, which may compromise the efficacy of standard-dose PPI-based eradication regimens. Studies have shown that extensive metabolizers, due to rapid PPI metabolism, are unable to maintain sufficiently high intragastric pH levels, thereby affecting antibiotic stability and bactericidal activity. Compared with poor metabolizers, extensive metabolizers have a 2.52-fold higher risk of eradication failure when using CYP2C19-dependent PPIs (Shah et al., 2021). Therefore, for patients in this region, alternative strategies such as using double-dose PPIs, selecting newer acid suppressants like potassium-competitive acid blockers (P-CABs), or prolonging treatment duration may be considered to improve efficacy. In summary, given the predominance of extensive and intermediate metabolizer phenotypes in the Nanjing population, the impact of patient metabolic phenotype on PPI efficacy should be carefully considered during treatment, and enhanced acid suppression strategies should be employed when necessary to improve eradication success rates.

Molecular biology techniques can be applied to invasively collected specimens such as gastric mucosa and gastric fluid, as well as to non-invasively collected specimens such as feces, saliva, and dental plaque, offering greater accessibility. The accuracy of molecular techniques is not affected by medications, and they can simultaneously detect *H. pylori* infection and antibiotic resistance, leading to their increasingly widespread use in the *H. pylori* field. PCR is the most commonly used molecular detection method, with advantages including the requirement for a small initial bacterial load in the sample and relatively low demands on equipment and technical expertise. Studies have shown that PCR for the diagnosis of *H. pylori* infection and the detection of clarithromycin and levofloxacin resistance genotypes in gastric mucosal samples achieves sensitivity and specificity exceeding 95% (Li et al., 2020; Zhao et al., 2022), which is highly consistent with our findings. Our study demonstrated Kappa values of 0.9046 for clarithromycin and 0.9187 for levofloxacin, both indicating excellent concordance and suggesting that genotypic testing accurately reflects the phenotypic resistance status for these two drugs. Therefore, using gastric mucosal samples combined with PCR, this study achieved reliable and highly accurate diagnosis of *H. pylori* infection and resistance to clarithromycin and levofloxacin, facilitating large-scale epidemiological investigations. Further analysis of resistance-associated gene mutations showed that among the 3,177 samples that completed *23S rRNA* gene testing, the A2143G mutation accounted for 98.93% of cases, representing the predominant molecular mechanism of clarithromycin resistance. The A2142G and A2143C mutations were extremely rare. This finding is consistent with previous reports from other regions (Tran et al., 2024; Martínez-Martínez et al., 2026), suggesting that testing for the A2143G site alone can cover the vast majority of clarithromycin-resistant cases. Among the 826 samples that completed *gyrA* gene testing, N87K, D91N, and D91G were the most common mutation types. Although compound mutations were diverse, their overall proportion was low.

Furthermore, the Kappa values for amoxicillin, tetracycline, furazolidone, and metronidazole were 0.0239, 0, 0.0005, and 0.1004, respectively, all indicating low and statistically non-significant concordance. This suggests that genotypic testing for these drugs cannot yet effectively replace phenotypic susceptibility testing, a finding that may be attributable to several factors. First, the resistance mechanisms for some antibiotics are complex. For example, metronidazole resistance involves mutations in multiple genes, including *rdxA*, *frxA*, and *frxB*, which may not be fully captured by current testing methods. For amoxicillin, resistance primarily involves mutations in the *PBP1A* gene encoding penicillin-binding protein 1A. However, other resistance mechanisms have also been reported, including mutations in *PBP2* or *PBP3*, reduced outer membrane permeability, and the production of β-lactamases. Our testing focused exclusively on *PBP1A* mutations, which may not detect all resistant strains. Second, the resistance mechanisms for certain drugs have not been fully elucidated. For instance, research on genes associated with resistance to furazolidone and tetracycline remains relatively limited. Third, the small number of phenotypically resistant samples for some drugs in this study may have affected the stability of the concordance assessment. Notably, although the Kappa value for metronidazole was only 0.1004, the genotypic test showed a high sensitivity of 99.61%, indicating its strong potential to identify metronidazole-resistant strains. However, its specificity was only 6.67%, indicating a large number of false-positive results where genotypic testing indicated resistance while phenotypic testing showed susceptibility. This may be related to non-specific mutations or the selection of testing targets in the metronidazole resistance mechanism. Future studies should focus on optimizing detection targets to improve specificity. Therefore, while genotypic testing for clarithromycin and levofloxacin is highly concordant with phenotypic results and suitable for clinical guidance, phenotypic antimicrobial susceptibility testing remains the gold standard for amoxicillin, tetracycline, furazolidone, and metronidazole. Future studies incorporating whole-genome sequencing and functional assays are needed to identify more reliable genotypic markers for these drugs.

Our multivariate analysis identified year as the only significant patient-level predictor of resistance for clarithromycin, levofloxacin, and metronidazole, with odds ratios of 1.096, 1.132, and 1.184 per one-year increase respectively. This finding quantifies the temporal increase in resistance rates and underscores the dynamic nature of antibiotic resistance in *H. pylori*. The lack of significant associations with age and sex suggests that demographic factors play a limited role in determining resistance risk in this cohort. These results support the need for regular regional resistance surveillance and guideline updates, rather than age- or sex-stratified treatment approaches.

This study has several limitations. First, as a single-center retrospective study, the sample source may be subject to selection bias. Second, the sample sizes for genotypic testing varied considerably across antibiotics, which may have affected the stability of the concordance assessment for some drugs. Third, this study did not include clinical treatment outcome data and therefore could not directly validate the actual therapeutic efficacy of genotype-guided therapy. Fourth, the genotypic testing panels for some drugs may not have covered all known mutation types, potentially leading to missed detection. Future studies incorporating multicenter prospective designs and clinical treatment follow-up are needed to further validate the value of genotype-guided individualized therapy. Fifth, this study included only 26 pediatric/adolescent patients (0.36% of the cohort), with 21 culture-positive isolates and 13 CYP2C19 genotyping results. While we have reported the resistance patterns and CYP2C19 phenotype distribution for this subgroup separately, the small sample size precludes definitive conclusions or robust statistical comparisons with the adult population. Therefore, our findings in pediatric patients should be considered exploratory, and larger prospective studies specifically designed for pediatric populations are needed to validate these observations. Sixth, a methodological caveat regarding our concordance analysis warrants mention. For antibiotics with very low phenotypic resistance rates in our cohort, particularly tetracycline with 0 resistant cases in the paired analysis of 1,345 samples, amoxicillin with 7 cases, and furazolidone with 1 case, the Kappa coefficient becomes statistically unstable and may be misleading when the number of positive cases is extremely small or zero. Therefore, the low Kappa values observed for these antibiotics, which were 0.0239, 0, and 0.0005 respectively, should not be interpreted as evidence of poor agreement between the two testing methods. They simply reflect the statistical limitation imposed by the rarity of phenotypic resistance. For these antibiotics, the very low resistance rates, namely 0.51% for amoxicillin, 0.07% for tetracycline, and 0.19% for furazolidone, are clinically reassuring, but they also mean that larger sample sizes are needed to validate genotypic markers. In contrast, for metronidazole, the number of resistant cases was sufficient with 1,286 cases, and the low Kappa value of 0.1004 likely reflects true poor concordance due to the complex, multi-gene nature of metronidazole resistance mechanisms. Future studies incorporating multicenter prospective designs and clinical treatment follow-up are needed to further validate the value of genotype-guided individualized therapy. Seventh, genotypic resistance testing in this study was performed on DNA extracted from the same biopsy specimens used for *H. pylori* culture. Therefore, only culture-positive samples were subjected to genotypic analysis. However, patients who were *H. pylori*-positive by histology or rapid urease test but culture-negative could potentially be tested using molecular methods that do not depend on viable organisms, such as PCR directly on biopsy specimens or stool samples. Future studies should explore the feasibility and accuracy of molecular resistance testing directly on culture-negative but histology-positive specimens.

## 5 Conclusion

This retrospective study of 7,227 *H. pylori*-infected patients in Nanjing between 2018 and 2024 systematically characterized the resistance profiles and molecular features of six commonly used antibiotics in this region. Resistance rates to metronidazole (84.45%), clarithromycin (50.01%), and levofloxacin (38.59%) were high, rendering these agents unsuitable for empirical therapy. In contrast, resistance rates to amoxicillin, furazolidone, and tetracycline were all below 0.6%, indicating that these drugs remain reliable treatment options. Annual trend analyses showed that resistance rates to metronidazole, clarithromycin, and levofloxacin generally increased over time, indicating a continuously growing resistance burden. Genotypic testing for clarithromycin and levofloxacin showed excellent concordance with phenotypic susceptibility (Kappa values >0.9), with resistance mutations highly concentrated at the A2143G locus of the *23S rRNA* gene (98.93%) and the N87K locus of the *gyrA* gene (52.54%), respectively, supporting their use for rapid clinical resistance detection. For metronidazole, concordance between genotypic and phenotypic testing was poor, which is likely attributable to the complex, multi-gene nature of metronidazole resistance mechanisms. For amoxicillin, tetracycline, and furazolidone, the extremely low number of phenotypically resistant isolates rendered the Kappa values statistically unstable. Therefore, these low Kappa values should not be interpreted as evidence of poor concordance. For these three antibiotics, phenotypic antimicrobial susceptibility testing remains the gold standard, and larger studies are needed to validate genotypic markers. CYP2C19 polymorphisms were predominantly of the extensive metabolizer (43.89%) and intermediate metabolizer (44.26%) phenotypes, highlighting the need for individualized acid suppression strategies. In summary, the resistance situation of *H. pylori* in this region is severe. It is recommended that regimens containing amoxicillin, furazolidone, or tetracycline be prioritized, and that genotypic testing be performed before using clarithromycin or levofloxacin to guide individualized treatment.

## Data Availability

The raw data supporting the conclusions of this article will be made available by the authors, without undue reservation.

## Supplementary material

Supplementary Table 1 Interpretive criteria for disk diffusion susceptibility testing of *Helicobacter pylori*.
