# Supplementary material for: Phenotypic and genotypic analysis of Helicobacter pylori resistance patterns, molecular characteristics, and CYP2C19 polymorphisms in Nanjing: a retrospective cross-sectional study
Source: Front Cell Infect Microbiol. 2026 Jun 23;16:1852534. doi: 10.3389/fcimb.2026.1852534 (PMC13337940; doi:10.3389/fcimb.2026.1852534)
Supplement: Supplementary Table 1 — Interpretive criteria for disk diffusion susceptibility testing of Helicobacter pylori. [file Table1.docx]

Supplementary Table 1 Interpretive criteria for disk diffusion susceptibility testing of *Helicobacter pylori*

| **Antibiotics** | **Types of interpretation** | | |
| --- | --- | --- | --- |
|  | Resistant | Intermediate | Sensitive |
| Clarithromycin | ≤13 mm | 14-17 mm | ≥18 mm |
| Levofloxacin | <13 mm | 13-17 mm | >17 mm |
| Amoxicillin | <14 mm | 14-17 mm | >17 mm |
| Furazolidone | ≤14 mm | 15-16 mm | ≥17 mm |
| Tetracycline | ≤14 mm | 15-18 mm | ≥19 mm |
| Metronidazole | <16 mm | 16-21 mm | >21 mm |
